# Steroids after the Kasai procedure for biliary atresia: the effect of age at Kasai portoenterostomy

**DOI:** 10.1007/s00383-015-3836-3

**Published:** 2015-11-21

**Authors:** Athanasios Tyraskis, Mark Davenport

**Affiliations:** Department of Paediatric Surgery, King’s College Hospital, Denmark Hill, London, SE5 9RS UK

**Keywords:** Biliary atresia, Kasai portoenterostomy, Adjuvant steroids, Prednisolone

## Abstract

The use of adjuvant steroids following Kasai porteoenterostomy (KPE) for biliary atresia is controversial. The aim of this study was twofold: a systematic review of published literature and an update of the clinical Kings College Hospital series to look for evidence of an effect of age on the outcome in a group of BA infants treated with high-dose steroids. This clinical study included infants treated between January 2006 and June 2014 who underwent KPE by day 70 of life and who received high-dose steroids (oral prednisolone starting 5 mg/kg/day). They were subdivided into cohorts according to age at which KPE was performed. The outcome measured was clearance of jaundice (<20 µmol/L) by 6 months and native liver survival. R × C χ^2^ analysis and log-rank tests were used, respectively, and *P* ≤ 0.05 was regarded as significant. 104 infants were included with a median age at KPE of 45 (range 12–70) days. 71/104 (67 %) cleared their jaundice by 6 months of age. Age-cohort analysis showed a trend (*P* = 0.03) favouring early KPE (e.g. 100 % of 11 infants operated on <30 days clearing their jaundice compared to 66 % of those operated on between 61 and 70 days). There was a significant native liver survival benefit for those operated on <45 days (5 year NLS estimate 69 versus 46 %; *P* = 0.05). Clearance of jaundice is related to the age at KPE in infants who receive high-dose steroids. Native liver survival appears to be improved as a result of this. This is the first study to show tangible longer-term benefit from high-dose steroids in biliary atresia.

## Introduction

Biliary atresia (BA) is an obstructive cholangiopathy affecting both intra- and extrahepatic ducts which present typically as persistent conjugated jaundice in the newborn. It is rare but with obvious geographical variation. So in Europe and North American it has an incidence of 1 in 15–20,000 live births [1–3] and by contrast can be seen in up to 1 in 5000 live births in Taiwan and presumably mainland China [4]. It is not a homogenous, uniform disease and within the umbrella term are several variants which have separate and distinct causes and different outcomes [5–8]. These make comparison of treatment options difficult but not impossible.

The key to success is to improve the outcome following Kasai portoenterostomy (KPE) and thereby retaining the native liver. This avoids all the immediate potential operative hazards of liver transplantation and the longer-term problems of immunosuppression such as post-transplant lymphoproliferative disease.

So how best to achieve this in practice? Assuming that there is surgical familiarity and experience with the operation then we have to look at the effectiveness of adjuvant medical intervention. There are a number of areas which might improve overall outcome. So, early post-operative cholangitis is detrimental to long-term outcome and can be seen in up to 50 % of surgical series. In the early years, surgeons came up with evermore fanciful intestinal variants on the simple Roux loop but none stood the test of time and a measured 40 cm length now appears standard and unchallenged. Various antibiotic regimens have been tested (although none in a randomized trial) and continuation beyond the early post-operative period does not appear to offer any greater protection. However, the main focus of attention during the past 15 years has been on the efficacy of steroids and the rest of this review will be devoted to an examination of that.

## Mechanisms of action

The mechanism by which steroids may provide benefit is not completely understood but studies have shown that there may be two potential modes of actions: increasing choleresis and bile flow, and an anti-inflammatory effect either systemically within the liver or locally on the anastomosis.

Karrer et al. [9] reported and quantified increased biliary production following administration of a short course of high-dose steroids (starting at 10 mg/kg methylprednisolone decreasing by half each day for a total of 4 days) in patients post-HPE who had either recalcitrant cholangitis or marked reduction of bile flow. They found that this “blast” therapy significantly increased mean bile volume production compared to controls (from about 135 to 158 mL/day) decreased mean serum bilirubin (from 102 to 84 μmol/L), but also lowered mean bile bilirubin concentration (from 21 to 16 mg/dL) [9]. Miner et al. described the choleretic effect of steroids in rats and showed that it is via the induction of Na^+^-K^+^ ATPase activity resulting in an increase in electrolyte transport and subsequently an increase of the total bile salt-independent fraction of bile [10, 11].

Steroids are potent anti-inflammatory drugs and there is evidence that at least in some infants with biliary atresia there is a marked inflammatory component. We have shown that in up to 50 % of a series of infants with otherwise isolated BA there is abnormal expression of HLA-DR antigen and the adhesion molecules ICAM and VCAM together with an infiltrate of immune-activated T cells (predominantly CD4^+^ Th1 and Th17) and NK cells in about 30 % [12–14]. Furthermore, there is a marked systemic inflammatory process post-KPE characterized by an increase in serum inflammatory cytokines (TNF-α, IL-2, IL-12) and soluble adhesion molecules which may be sustained up to 6 months post-surgery [15]. This on-going systemic inflammatory process and local intrahepatic inflammation is certainly a tempting therapeutic target whereby steroids could limit hepatocyte destruction and fibrosis.

## Early reports

Morio Kasai himself used steroids empirically and intermittently as part of the Sendai post-operative regimen although never published specifically on them or investigated their effect in isolation [16]. Karrer and Lily published their experience with the use of steroids in 1985 in those with recalcitrant cholangitis or sudden cessation of bile flow and began the concept of “blast therapy” [9]. Thereafter Muraji et al. from Kobe, Japan explored dosage and length in a variety of small uncontrolled reports [17]. An influential (at least in the USA) uncontrolled single-centre series on high-dose steroids (intravenous then oral methylprednisolone) and long-term antibiotics was reported by Meyers et al. from Salt Lake City [18]. An unprecedented clearance of jaundice of almost 80 % (11/14) was reported in the steroid group compared to a very poor 21 % (3/14) in the non-steroid group. This translated into much improved native liver and overall survival rates.

## Non-randomized evidence

Stringer et al. [19] reported the outcome of 50 infants treated with dexamethasone (starting at 0.3 mg/kg bd for 5 days) and achieving a 70 % clearance of jaundice compared with a smaller non-steroid group (*n* = 10) who only managed a clearance of 40 %.

Escobar et al. [20] published a retrospective case series with 43 children (21 received steroids and 22 untreated controls) and found that the treated group had significantly higher levels of clearance of jaundice at 6 months (76 versus 37 %, *P* = 0.01), lower rates of transplantation (37 versus 63 %, *P* = 0.2), but similar rates of survival (86 versus 82 % control). However, in this study dose, duration and steroids used varied considerably and were determined according to the surgeon’s preference.

Kobayashi et al. [21] reported the first retrospective trial for altering steroid dosage dependent on stool appearance. This retrospective case series compared five different patient groups: no steroids (*n* = 12); three groups of patients with a weaning prednisone regimen starting at doses from 6, 10, and 20 mg, respectively, and total course lasting 9, 9, and 15 days, respectively, and a final weaning group starting at 20 mg but who used stool colour to guide duration and dose. If stools began to turn pale, the steroid dose was increased back to 20 mg/day in an attempt to increase bile flow. They found that the stool-colour guided group had significantly higher rates of infants who became jaundice-free (91 versus 58 % without steroids), time to become jaundice-free was significantly shorter (33 versus 83 days without steroids) [24]. They did not report patient weights and it is likely that the 20 mg group was the only one to have taken a high dose of steroid and thus this trial cannot be directly compared to others.

These usually favourable small-scale reports were tempered by a prospective single-centre study from Hannover, Germany who used an open-label regimen of “high-dose” steroids in 20 consecutive infants and compared to 29 historical controls [22]. The format combined intravenous methylprednisolone at a high dose (10 mg/kg day 1–5) but then a much reduced dose of 1 mg/kg from day 6 to 28. Unfortunately, the results in either group were poor and only 27 % of the entire group were still jaundice-free at 2 years post-KPE.

Shimadera et al. [23] reported 23 cases treated with 3–5 mg/kg/day of prednisolone from the 7th post-operative day until jaundice cleared. They found that there was a significant impact on mean steroid dose on the post-operative jaundice period (shorter post-operative jaundice for higher doses, correlation coefficient 0.48, *P* = 0.02). They did not report long-term follow-up and thus cannot be easily compared to other studies.

A large retrospective study [24] from Shanghai, China of 380 infants was published by Dong et al. in 2013. It compared outcomes of infants receiving a high-dose regimen (IV methylprednisolone at 4 mg/kg tapered to completion with oral prednisolone) or a low-dose regimen (starting at 4 mg/kg but tapered quickly for 1–2 weeks). There was a significant difference in age at KPE (74 versus 66 days, *P* = 0.03) favouring the high-dose group. There was a greater proportion of infants clearing jaundice to normal at 3 months in the high-dose group (53 versus 39 %, *P* = 0.007).

## Randomized trial evidence

The first randomized, placebo-controlled, double-blinded study included infants (*n* = 71) from two UK centres and used a low-dose 6-week course of oral prednisolone starting at 2 mg/kg/day [25]. There was a significant reduction in bilirubin levels at 1 month which was more obvious in those who underwent KPE at <70 days (66 versus 92 μmol/L, *P* = 0.06) [24]. There was no significant effect on clearance of jaundice at 6 months (47 versus 49 %) or subsequent native liver survival.

The follow-up study published in 2013 then included 44 further infants treated with high-dose prednisolone starting at 5 mg/kg/day and found that there was now a significant difference in clearance of jaundice when comparing the total steroid group (low and high dose) to the placebo group (52 versus 66 %, *P* = 0.037) [26]. However, there was no significant improvement to overall survival (95 versus 96 %) or native liver survival (56 versus 48 %) when comparing the high-dose group to no steroids. This study also found that there were significantly lower levels of bilirubin (58 versus 91 μmol/L, *P* = 0.002), AST (118 versus 155 IU/L, *P* = 0.002), AST to platelet index (APRi) (0.49 versus 0.82, *P* = 0.005), but not γGT (1250 versus 1000, *P* = 0.11) in the high-dose steroid group compared to placebo or controls.

The North American START (STeroids in biliary Atresia Randomized Trial) randomized placebo-controlled trial studied a high-dose steroid regimen (IV methylprednisolone starting at 4 mg/kg/day on day 1 post-KPE) for 2 weeks then oral prednisolone at 2 mg/kg/day and tapering down for 9 weeks in 70 infants [27]. They found that there was a non-significant improvement in clearance of jaundice at 6 months (59 versus 49 %), which was higher in the sub-group coming to KPE at <70 days (72 versus 57 %) [4]. Results from this trial are compared to Davenport et al. 2013 in Table 1, as they are currently the highest quality studies performed to date.

**Table 1:** Comparison between largest randomized trials

	START [27]	Kings College Hospital Trial [26]
Design	Randomized, placebo-controlled (2005–11)	Randomized placebo and open-label (2000–11)
Centres	18	Single surgeon
Controls	70	91
Subjects	70	Low dose (*n* = 17)
		High dose (*n* = 44)
Steroid regimen	IV MP d1–d4	Low: oral P, d7–d21 (2 mg/kg/day) then d22–d28 1 mg/kg/day)
	Oral P d5–d13 (4 mg/kg/day)	
	Oral d14–d91 (2 mg/kg/day)	High: oral P, d5–9 (5 mg/kg/day) then d10–d14 (4 mg/kg/day) etc. to d30
Entry	Isolated, BASM, CMV unaware	Isolated, CMV –ve
Age at KPE	Mean 69 days	Median 50 days (all <0 days)
Main outcome measure	<25 µmol/L @ 6 months	<20 µmol/L @ 6 months
Outcome		
Overall	41/70 (57 %) versus 34/70 (49 %), *P* = 0.43	
Age <70 days	*n* = 76	
	28/39 (72 %) versus 21/37 (57 %), *P* = 0.36	41/62 (66 %) versus 47/91 (52 %), *P* = 0.037

*(M)P* (methyl)prednisolone

It is likely that the true difference in clearance of jaundice levels at 6 months between a steroid treatment group and control group is closer to 10 % as was found by Davenport et al. in 2013 [26] and Bezerra et al. in 2014 [27] rather than greater differences as reported by Meyers et al. [18] When comparing the two largest prospective or RCT trials which investigated the effect of high-dose steroids versus no steroids, a nearly identical relative risk was found for clearance of jaundice at 6 months (1.23 in Bezerra et al. versus 1.28 in Davenport et al.) [26, 27].

The Japanese Biliary Atresia Society conducted a multicenter randomized trial [28] of post-operative steroid treatment comparing different doses of prednisolone; one group starting at 4 mg/kg/day and having 52 mg/kg in total over 30 days (*n* = 35), and the second group starting at 2 mg/kg/day and having a total of 29 mg/kg over the same period (*n* = 34). They found that there was a significantly lower bilirubin level for the patients in the high-dose group both at 1 month (37 versus 58 µmol/L, *P* = 0.03) and 2 months (22 versus 37 µmol/L, *P* = 0.03). No other significant differences were found in other liver function tests or complication rates but long-term outcomes were not reported.

In summary, there is level 1A evidence for the beneficial effect of high-dose steroids in increasing rates of clearance of jaundice by 6 months. Current evidence does not support beneficial long-term effects on native liver survival or overall survival, with only 3B of evidence supporting the use of steroids.

## Systematic review and meta-analysis

Lao et al. used hospital coding data and identified 516 children across the USA treated by KPE between 2003 and 2008 [29]. Of these 239 (46 %) received steroid post-operatively. There appeared to be no demographic differences between those prescribed steroids and those not. The only outcome measure studied was length of stay and this favoured the steroid group, by about 3 days. Still the mean length of stay overall was 14 days, which seems inexplicably high in any case. Characteristic of American experience over half of all infants underwent surgery at >60 days.

A nationwide survey from Japan combined data from heterogeneous regimens for duration, doses, and rout of administration of steroids and performed a sub-group analysis in type III BA (obstruction at the porta hepatis) and compared low-dose (<4 mg/kg) and high-dose (≥4 mg/kg) prednisolone groups to a steroid-free group [30]. They found that overall the steroid group had a non-significant higher rate of native liver survival (58 versus 35.7 %, *P* = 0.05), but when comparing the high-dose group to no steroids, there was a significant increase in native liver survival (58.9 versus 35.7 %, *P* = 0.05). However, wide-spread acceptance of the use of steroids in BA in Japan meant that there were only 14 patients in the no steroid group and 208 who did have steroids.

**Fig. 1:**
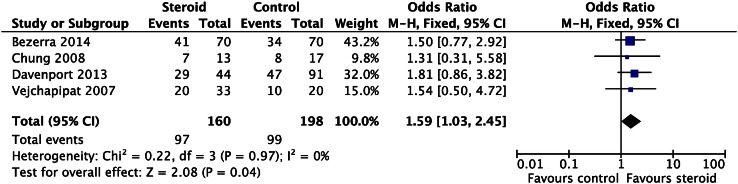
Systematic analysis: effect of high-dose steroids on clearance of jaundice (aged <70 days at Kasai portoenterostomy). (Reproduced from Chen et al. [33])

There have been three meta-analyses [31–33] published to date with varying entry criteria for analysis. The most recent meta-analysis to be published included studies to 2014 [randomized controlled trials (*n* = 2) and observational studies (*n* = 5)] with a total of 259 cases. They found that there was a significant difference in clearance of jaundice at 6 months (OR 1.59, 95 % CI 1.03–2.45, *P* = 0.04), in patients treated with high-dose steroids, particularly if <70 days at surgery [33] (Fig. 1).

This report and other trials emphasise the major problem with studies in rare diseases. In order to detect a 10 % difference in jaundice-free proportions for instance at 6 months between two groups of equal size using the conventional power of 80 % (which allows therefore for a 20 % chance of a type II error of not detecting a true difference) and an alpha of 5 % then 387 cases are needed for each group [34]. The START trial set out to try and identify a clinically important difference of 25 % [27]. In retrospect, this appears unduly optimistic and was not based on previous studies other than perhaps that of Meyers et al. [18] where only 21 % of a control non-steroid group was able to clear their jaundice. Realistically, these numbers will not occur outside of a huge international multi-centre trial.

## Complications

Most studies have not systematically been reporting complications of steroid treatment. Some have investigated if there is a difference in cholangitis rates between the treatment groups occasionally finding a significant improvement in rates of cholangitis in the steroid group. Two studies have found decreased rates of cholangitis when comparing high to low-dose steroids. Dong et al. [24] reported that rates of cholangitis by 1 year were significantly lower in the high-dose steroid group when compared to low-dose steroids (32 versus 48 %, *P* *<* 0.01) and there was no significant increase of morbidity associated to the higher dose steroids.

In the literature, there have been specific cases with rare complications such as ‘fluid retention’, a gastrointestinal bleed associated to a rotavirus enteritis in a patient taking dexamethasone, and one case of cytomegalovirus meningitis with high-dose steroids [17, 19].

The START trial did systematically report serious adverse events and did not find an overall difference in the number of events in the steroid compared to the control group (81 versus 80 %) but did find that adverse events occurred earlier [27]. Thus, time of onset of their first serious event was defined as by the first 30 days post-KPE (37 versus 19 %; *P* = 0.008). No serious adverse events attributable to the steroids were reported in either UK study [25, 26].

There is some disconcerting evidence of adverse effects from use of high-dose dexamethasone in extremely low-birth-weight preterm infants (e.g. spontaneous gastrointestinal perforations, smaller head circumference, and delayed cognitive development) [35, 36]. However, these are clearly not the usual infants treated for biliary atresia, being older, and actual evidence is lacking. Only long-term prospective study will establish safety of any steroid therapy.

There is currently level 1B evidence that high-dose steroids do not significantly increase rates of adverse events but that adverse events can occur earlier than patients who do not take steroids.

## The effect of age on efficacy of high-dose steroids

The actual age at which infants undergo KPE appears to be important in terms of response to steroid. This point was emphasised in our original randomized study [25] and we chose to present our next set of data limited to only those infants who were <70 days at time of KPE [26]. This was the subject of a separate sub-group analysis in the START trial although still the difference never achieved statistical significance [27]. By contrast, a difference was shown in the Japanese multi-centre trial attributable to age at KPE [28].

Our single-centre prospective, now open-label, trial has continued to recruit infants and further results from this are now presented with the aim of showing that there is a specific effect of age on outcome using adjuvant steroids.

### Methods

This study includes only infants who received high-dose steroids (regimen as previously described). All underwent KPE by day 70 of life. All infants were followed prospectively. Post-operative medical regimens were standardized as far as practical and most infants received the same medium-chain triglyceride-enriched formula feeds (Caprilon, SHS) with added calorie supplements (Duocal©powder, SHS) providing approximately 120–150 kcal/kg/day; intravenous antibiotic prophylaxis (Tazocin© and gentamicin) for 5 days, followed by oral cefalexin (25 mg/kg/day) for 28 days. Cholestyramine (1/2 sachet bd) was also given until clearance of jaundice (if achieved). Long-term medications included phenobarbitone (15 mg/day) and oral fat-soluble vitamin supplementation.

Age-cohort analysis divided infants on the basis of age into discrete cohorts (<30 days, 31–40 days, etc.) and calculated for each the proportion of those to clear their jaundice (<20 µmol/L) by 6 months of age [37]. The cumulative probability was calculated by adding successively older cohorts. Differences both absolute and for trend were assessed using an R × C Chi square test. Kaplan–Meier survival curves were calculated for two groups based upon the median age at surgery and compared using log-rank tests. A *P* value of ≤0.05 was regarded as significant.

### Results

The cohort comprised 104 infants with a median age at surgery of 45 (range 12–70) days and treated between January 2006 and June 2014. Within this cohort were those with isolated BA (*n* = 80), BASM (*n* = 9), cystic BA (*n* = 10) and CMV IgM+ve BA (*n* = 5).

**Fig. 2:**
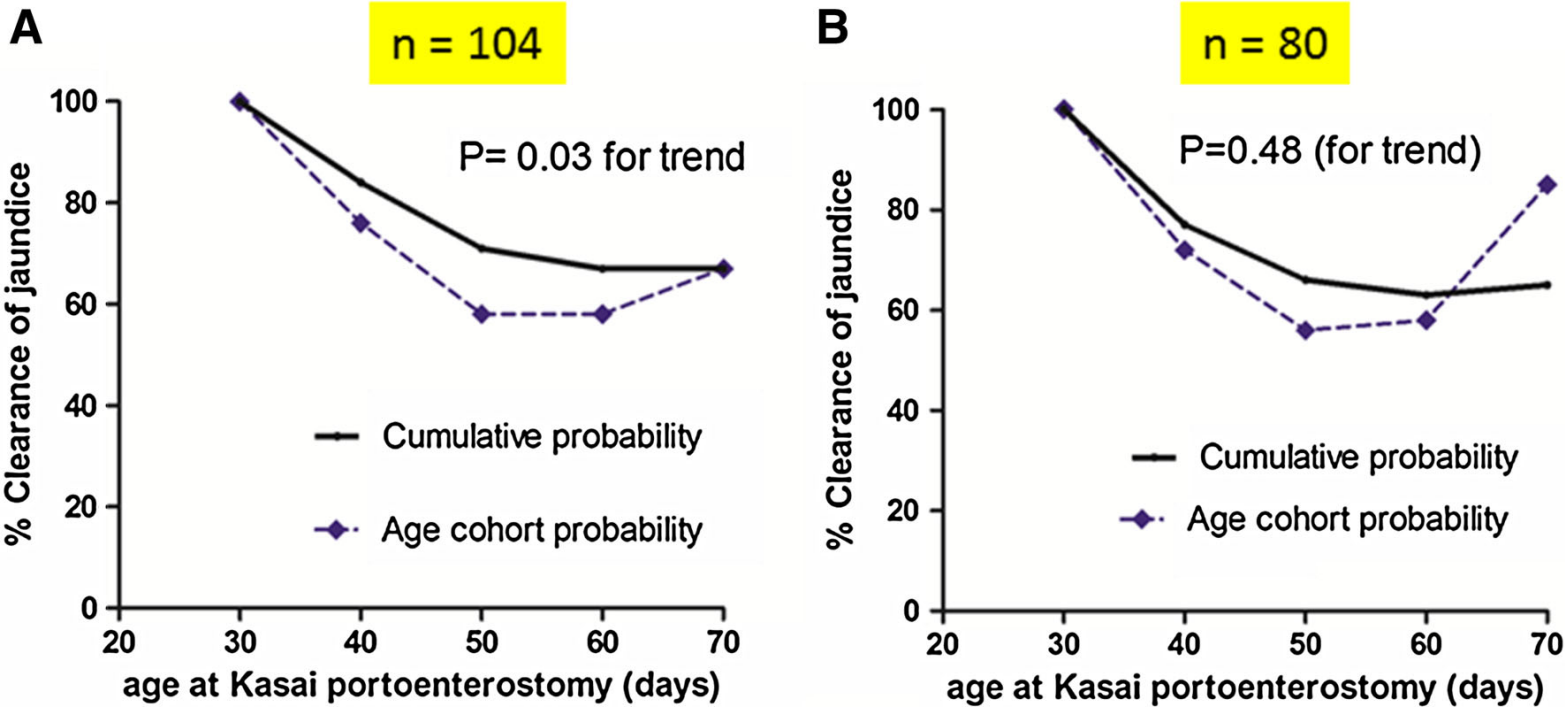
2006–2014 Kings College Hospital (infants ≤70 days). Age-cohort analysis: percentage clearance of jaundice at 6 months for infants defined by age at Kasai portoenterostomy (*n* = 104) (**a**) and for isolated biliary atresia alone (**b**)

Overall 71/104 (68 %) cleared their jaundice by 6 months of age. Age-cohort analysis showed a significant trend over time favouring early HPE (*χ*^2^ = 7.6, *P* = 0.10 and for trend *χ*^2^ = 4.5, *P* = 0.03) (Fig. 2). This is reinforced by the observation that all 11 infants <30 days at KPE cleared their jaundice compared to 67 % of those in the 61–70 day cohort. The falling cumulative line illustrates this concept in Fig. 2a.

A sub-group analysis was performed using only those with otherwise isolated BA (*n* = 80) and is illustrated by Fig. 2b. There was no overall effect (*P* = 0.2) nor for trend (*P* = 0.48) in this analysis.

**Fig. 3:**
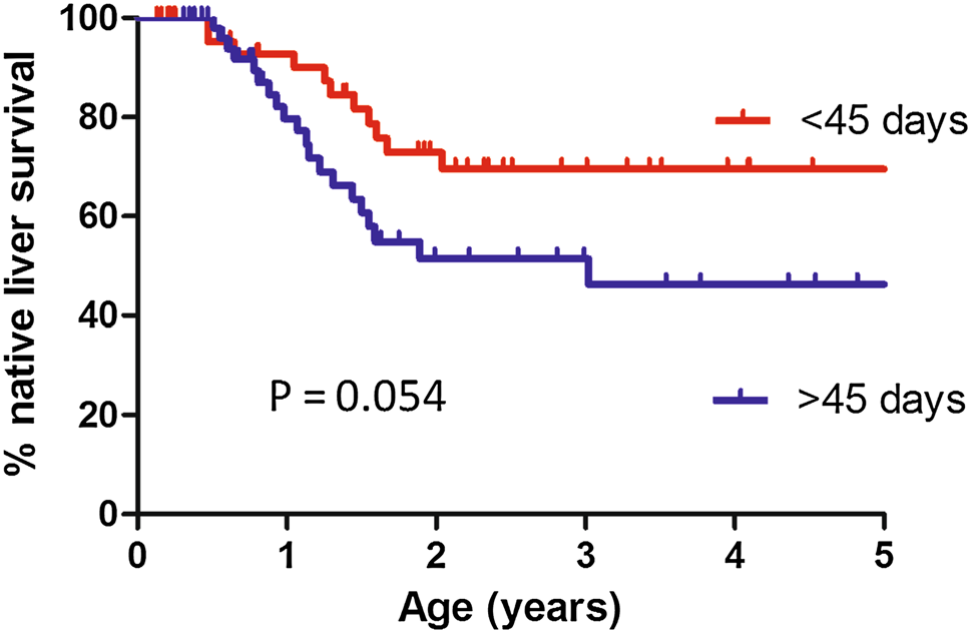
Kaplan–Meier curve of native liver survival over time comparing HPE before or after 45 days of age in patients who received high-dose steroids

Overall native liver survival is illustrated by Fig. 3, divided according to the median age at KPE for the group (45 days). There was a significant survival advantage for those operated on <45 days of life which reached statistical significance (5 year NLS estimate 69 versus 46 %; *χ*^2^ = 3.7, *P* = 0.054).

### Discussion

This series and analysis strongly suggests a marked effect of age on the efficacy of high-dose steroids on the main clinical outcome following KPE (clearance of jaundice) in infants with biliary atresia, in general. Thus, the falling cumulative probability of clearance appeared real in our series of over 100 infants. However, trying to prove this for the main grouping of isolated BA proved difficult and removing the two developmental groups (BASM and CBA) abolished any statistical significant relationship even though the graphical pattern was very similar.

**Fig. 4:**
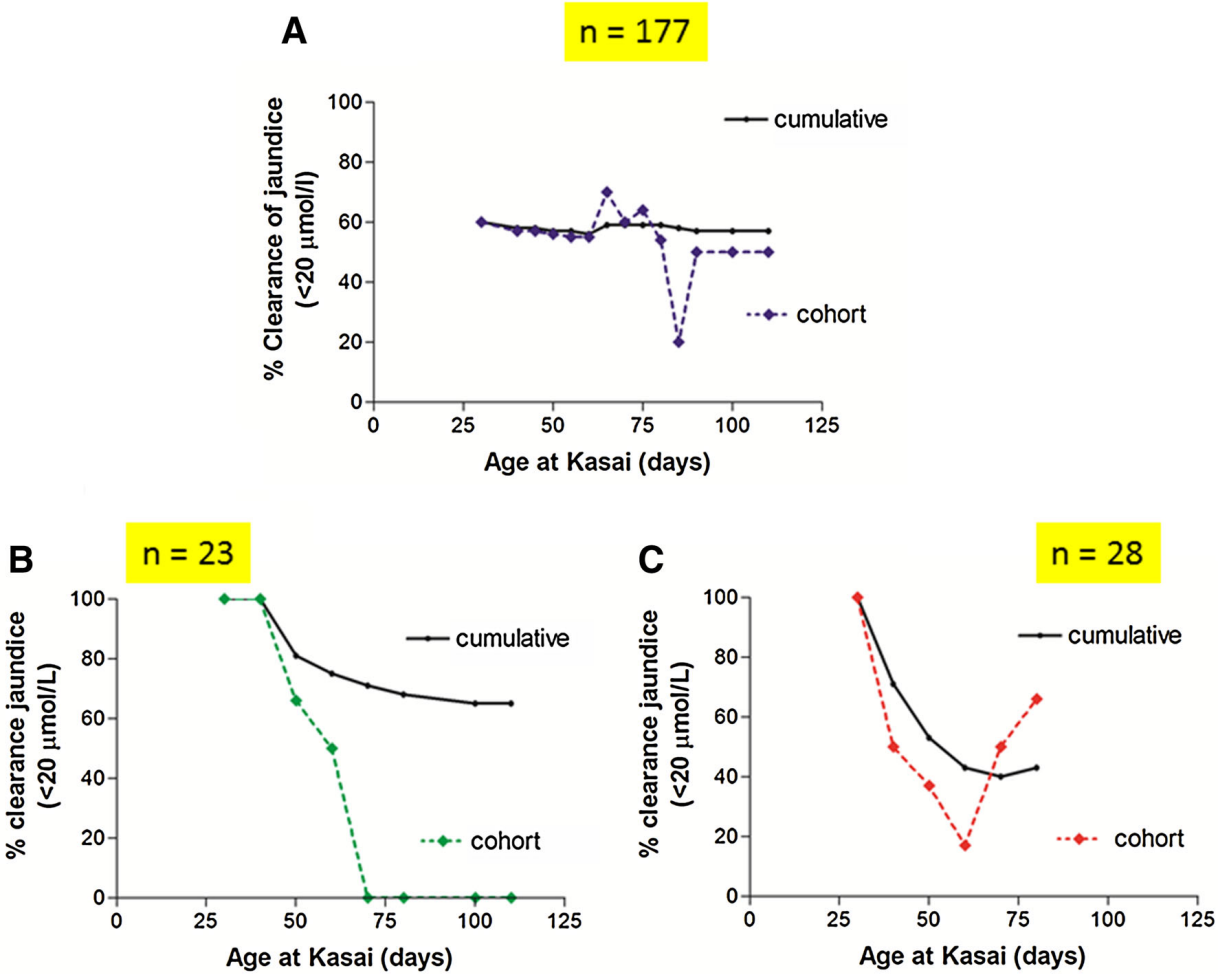
1994–2005 Kings College Hospital cohort (all ages): clearance of jaundice (<20 μmol/L) by age cohort and cumulatively for isolated biliary atresia (*n* = 177) (*χ* ^2^ = 6.7, *P* = 0.75, for trend *P* > 0.9) (**a**), cystic biliary atresia (**b**) (*n* = 23) and BASM (**c**) (*n* = 28). The latter two groups were combined as “developmental biliary atresia” and then there was a significant difference both overall (*P* = 0.02) and for linear trend (*P* = 0.02). (Figures derived with permission from reference Davenport et al. [37])

Nonetheless, we believe that the effect to be real when we compared the current results to our original published study using age-cohort analysis in a large cohort of infants (*n* = 225) treated in our institution from 1994 to 2005 [37]. The vast majority (>95 %) of those infants did not have any adjuvant steroids (though some were part of the low-dose steroid trial) and their clearance rate for the cohort was 56 % compared to 68 % currently. We showed that only those with cystic BA and BASM had any kind of relationship of outcome with the age at KPE and in the large group with otherwise isolated BA the cumulative probability line was resolutely flat (Fig. 4).

## Conclusions

Current evidence based on the most recent systematic review of published evidence supports the view that high-dose steroids do have a significant benefit in reduction of post-operative bilirubin and clearance of jaundice as the most frequently measured indices of the condition. We have shown that other biochemical markers indicating more specific liver injury (i.e. AST, APRi) are also affected by high-dose steroids at least in the first 6 months post-KPE, implying an actual effect on the underlying pathology of the disease process and not just perhaps on degree of restored bile flow.

This together with our current analysis of the King’s series suggests that there is a further reason to reduce the overall age at KPE—in that it improves the efficacy of adjuvant therapy. The implication is that the effect of steroids may be limited or inhibited by an increasing degree of fibrosis and onset of cirrhosis.
